# Corrosion Assessment of Myo-Inositol Sugar Alcohol as a Phase Change Material in Storage Systems Connected to Fresnel Solar Plants

**DOI:** 10.3390/molecules24071383

**Published:** 2019-04-09

**Authors:** José Miguel Maldonado, Ángel G. Fernández, Luisa F. Cabeza

**Affiliations:** GREiA Research Group, INSPIRES Research Centre, Universitat de Lleida, Pere de Cabrera s/n, 25001 Lleida, Spain; jmmaldonado@diei.udl.cat (J.M.M.); angel.fernandez@diei.udl.cat (Á.G.F.)

**Keywords:** corrosion, phase change material, myo-inositol sugar alcohol, Fresnel solar plant

## Abstract

Thermal energy storage systems work in conjunction with solar technologies with the aim of increasing their dispatchability and competitiveness in the energy market. Among others, latent heat thermal energy storage systems have become an appealing research subject and many efforts have therefore been invested in selecting the best phase change material (PCM) to fit the final application. In this study, an extended corrosion characterization was performed for two PCM candidates, solar salt (40 wt.% KNO_3_/60 wt.% NaNO_3_) and myo-inositol (C_6_H_12_O_6_), to be applied in Fresnel solar plants. Corrosion rates were determined in aluminium, stainless steel (AISI 304), carbon steel (AISI 1090), and copper by gravimetric tests, gauging the weight loss after 2000 h of immersion at 250 °C. The corrosion products were characterized by scanning electron microscopy (SEM) and x-ray diffraction (XRD). The corrosion tests carried out with myo-inositol did not show accurate enough results to draw conclusions regarding corrosion on the metals. However, it was observed that this sugar alcohol strongly sticks to the metal specimens, making myo-inositol extremely difficult to manage as an energy storage material. Therefore, the present paper discourages the use of myo-inositol as a PCM beyond its corrosion rate.

## 1. Introduction

According to the Global Risks Report 2018, failure of climate change mitigation and adaptation is among the top five global risks [1]. Reducing the greenhouse gas emissions by producing decarbonized energy is one of the European Union’s main objectives. Consequently, solar energy has developed considerably to tackle the greenhouse effect. By 2030, concentrated solar thermal energy, specifically, has been called upon to supply up to 7% of the world’s energy demands. This number will grow to 25% by 2050 if concentrated solar thermal energy efficiency continues to develop [2].

One of the main concerns regarding solar thermal technologies is the need to improve their competitiveness towards conventional power plants. To achieve that goal, reducing their levelized cost of electricity (LCOE) and enhancing their dispatchability are issues to be addressed, and concentrated solar power (CSP) plants are the most feasible option to provide power to the grid according to energy demands [3]. 

Regarding thermal energy storage systems, three different technologies are pursued by researchers: Sensible heat, latent heat, and thermochemical energy storage. Among those, latent heat storage using phase change materials (PCM) provides an efficient solution due to its high energy storage density [4].

Researchers in the solar energy field are focused on lowering the operational and maintenance costs of solar plants. Hence, the selection of proper storage materials is an important task, regarding not only the direct cost of the material but also the storage shell and safety issues. Several phase change materials have been studied at different working temperature ranges, including carefully selected thermal characterizations. The latter studies were developed mainly by differential scanning calorimeter (DSC), thermal gravimetric analysis (TGA), coupled with a quadrupole mass spectrometer (QMS) [5], polarized light microscopy and rheological measurements [6], or thermal cycling tests to check the aging of the PCM. Additionally, health hazard studies were developed by Miró et al. [7], Gasia et.al [8] and Maldonado et al. [9].

Considering storage materials have to be contained in a tank or even pumped in some CSP plants (with a two molten salts tank system), material characterization must be completed with a corrosion study. Corrosion tests are required to ensure the integrity of the facility and extend the life of the plant, as well as to reduce the operational budget as much as possible. The correct selection of materials for the tank, pipes and other items is a key parameter when designing a plant as the material costs are directly related to its capacity to resist corrosion. Consequently, the power plant’s LCOE can be lowered. There is a large list of available metals which can be used to build the storage tanks. Regarding alloys, the list contains nickel-iron alloy, stainless steel and carbon steels, listed from highest to lowest resistance to corrosion. Additionally, copper and aluminium are considered suitable choices for piping materials. From the alloy list, nickel-iron alloys are rarely chosen for current CSP technology due to their high cost. Several stainless steels were put through corrosion tests, as well as different carbon steel alloys in which the chromium percentage was varied [10], using solar salt (60 wt.% NaNO_3_ + 40 wt.% KNO_3_), the molten salt currently used as a storage material in CSP plants. Fernandez et al. [11] studied the corrosive effects of solar salt on stainless steel (AISI 304, 430) and on low-Cr steel (2.25 % Cr) at 390 ºC and at 550 °C. The samples were analysed by gravimetric analysis. As a result of this study, only the stainless steel 304 overcame the corrosion process. Also, Fernandez et al. [12] carried out a similar corrosion test on stainless steel 347, which is one of the most commonly used materials in CSP plants, immersing the steel in solar salts doped with nanoparticles (Al_2_O_3_ or SiO_2_). The procedure followed was the same as that of Reference [11], using gravimetric tests, scanning electron microscopy (SEM) and x-ray diffraction (XRD) to detect the resulting corrosion, producing favourable findings. Dorcheh et al. [13] also tested the immersion of alloys in solar salts. The chosen alloys were two ferritic steels—which did not withstand corrosion—two stainless steels (316 and 347H) and an NI-alloy (IN625), with the latter three materials showing satisfactory corrosion rates. In order to determine the corrosion rates, Dorcheh et al carried out gravimetric tests, followed by SEM analysis and electro probe micro-analysis (EPMA). It can be observed that the latter research studies were focused on solar salts (used as sensible heat storage) at temperatures higher than 500 °C. Several corrosion studies were carried out on materials with a final goal to work as a PCM. However, such studies were developed at low temperatures (up to 70 °C) [14,15,16]. In the current literature regarding the corrosion rates of phase change materials in CSP applications, there is a gap when temperatures of up to 300 °C are reached.

With the aim of developing a thermal energy storage (TES) system that works in conjunction with a linear Fresnel CSP plant at a working storage temperature of 250 °C, there are two suitable materials at this temperature range when considering the thermophysical properties: Solar salt and myo-inositol (C_6_H_12_O_6_) (Table 1) [9]. These two compounds were selected from a list of nine PCM candidates with melting temperatures between 210 °C and 270 °C. The selection was made based on the thermophysical properties defined by Maldonado et al. [9], and are summarized in Table 1. The current study completes the characterization of both materials by carrying out a corrosion test in contact with copper, aluminium, stainless steel (SS304), and carbon steel (AISI 1090).

## 2. Results and Discussion

### 2.1. Corrosion Produced by Solar Salt

**Figure 1:**
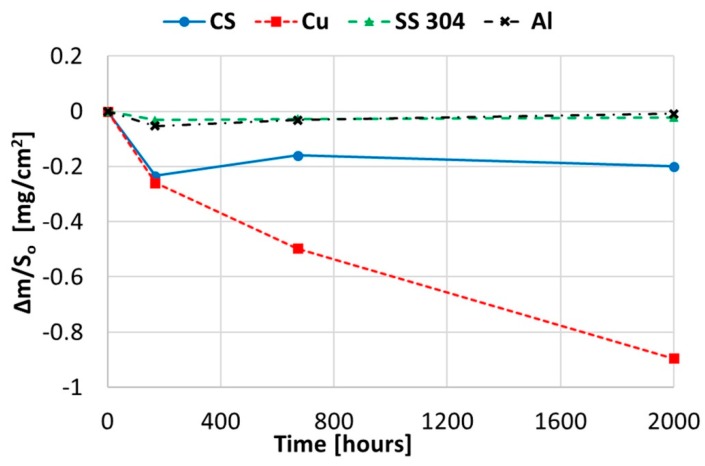
Gravimetric weight loss curve obtained for copper, aluminium, and stainless and carbon steel in solar salt at 250 °C.

Figure 1 shows the gravimetric weight loss chart obtained when immersing copper, aluminium, and stainless and carbon steel in solar salt at 250 °C for 2000 h. Stainless steel and aluminium can withstand the corrosion since they did not show weight loss at 250 °C. However, carbon steel and copper showed opposite results. Regarding carbon steel, the corrosion suffered during the 2000-h test was almost steady, demonstrating a corrosion rate of 0.01112 mm/year. For copper, the results showed a decreased capacity to withstand corrosion, and considering that the gravimetric curve will not continue to drop, the corrosion rate was 0.04437 mm/year. Nevertheless, it has to be taken into account that the trend showed by copper in Figure 1 could signify even worse results at longer periods.

**Figure 2:**
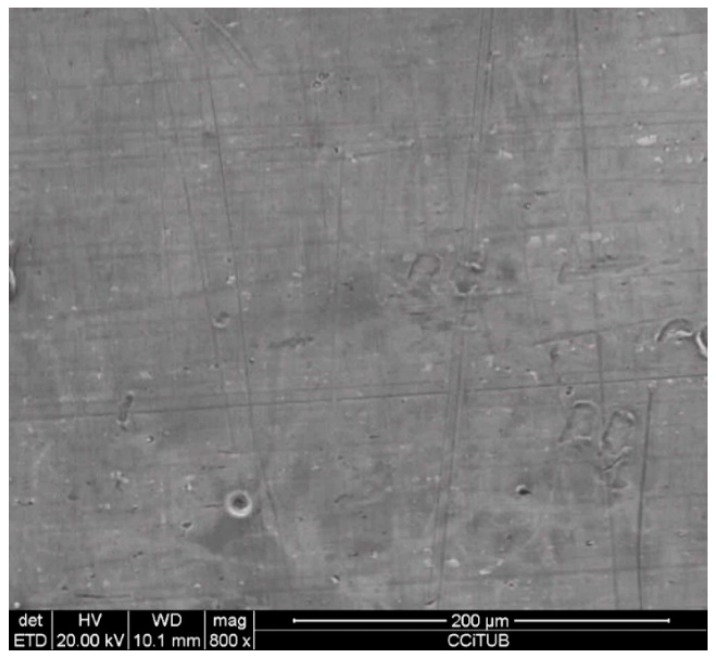
Detail of the aluminium specimen surface after 2000 h immersed in solar salt.

**Figure 3:**
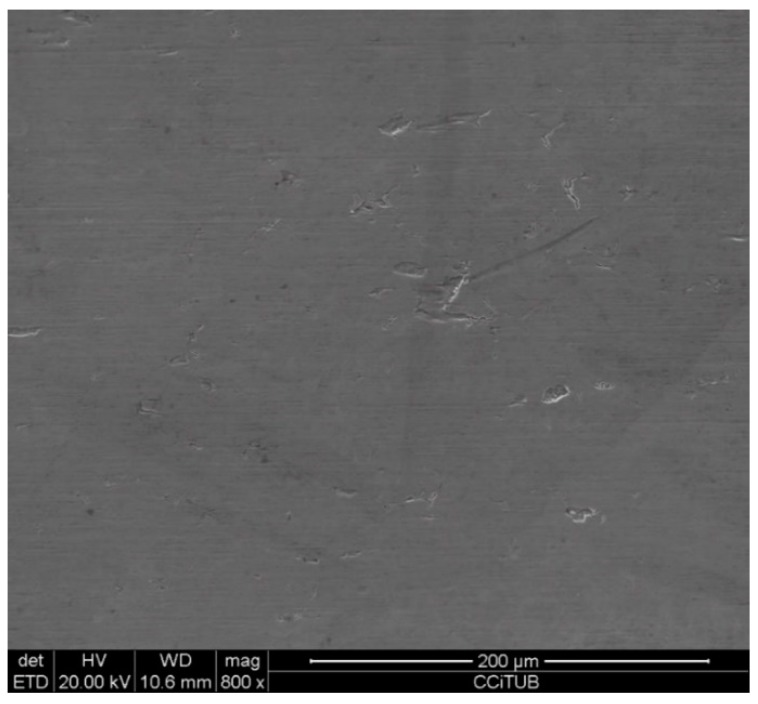
Detail of the stainless steel SS304 specimen surface after 2000 h immersed in solar salt.

**Figure 4:**
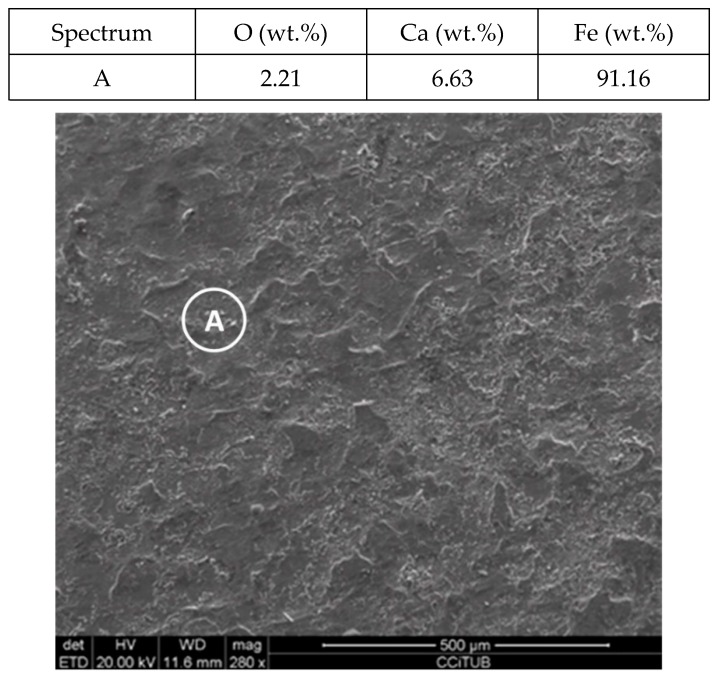
Detail (lower) and energy-dispersive X-ray (EDX) analysis (upper) of the carbon steel (AISI 1090) specimen surface after 2000 h immersed in solar salt.

**Figure 5:**
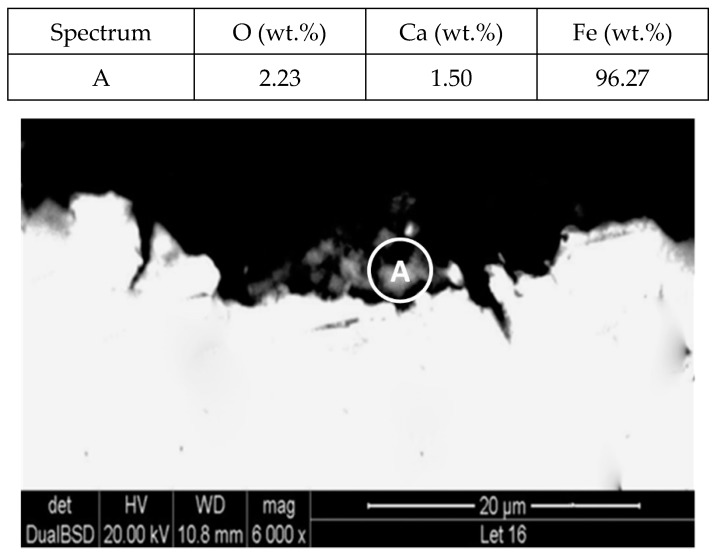
Detail (lower) and EDX analysis (upper) of the carbon steel (AISI 1090) specimen cross-section after 2000 h immersed in solar salt.

**Figure 6:**
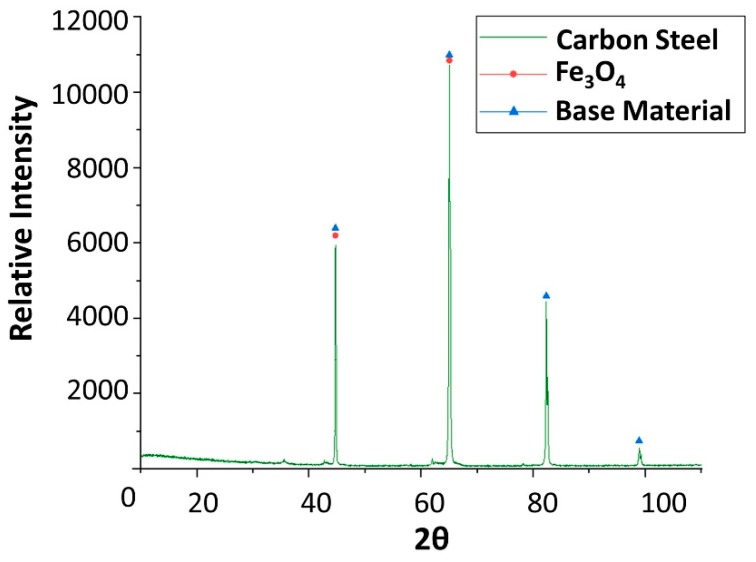
X-ray diffraction analysis of the carbon steel (AISI 1090) specimen after 4 weeks immersed in solar salt.

**Figure 7:**
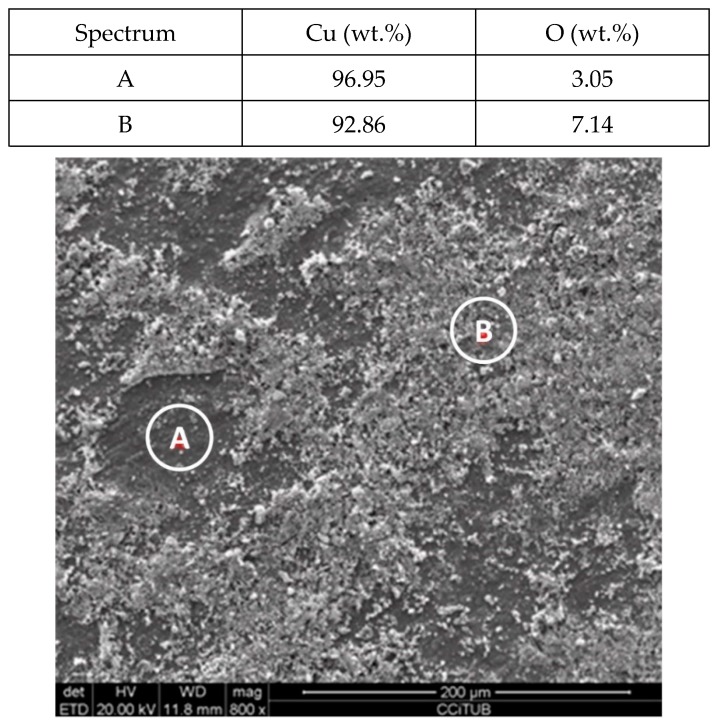
Detail (lower) and EDX analysis (upper) of the copper specimen surface after 2000 h immersed in solar salt.

**Figure 8:**
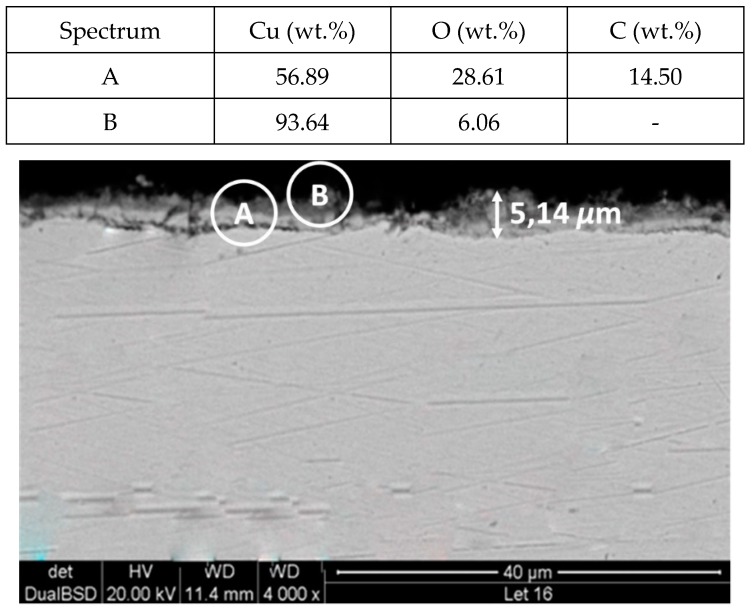
Detail (lower) and EDX analysis (upper) of the copper specimen cross-section after 2000 h immersed in solar salt.

To explain these results, a micro-structural (surface and cross section) study of the four coupons using scanning electron microscopy (SEM). These analyses were completed on the probes taken out of the crucibles after 2000 h, without performing any cleaning processes—unlike in the gravimetric study. Figure 2 and Figure 3 show the specimen surface morphology of aluminium and SS304, respectively, after 2000 h of immersion in stainless steel. As it was foreseen based on the gravimetric study, the samples were not corroded. Figure 4 depicts the carbon steel coupon surface, showing a layer composed of Fe, O and Ca (an impurity present in the salt) covering the sample. Subtle corrosion effects can also be recognized in the cross-section sample (Figure 5), wherein a non-protective corrosion layer of oxygen and calcium was obtained. The corrosion products were confirmed by XRD (Figure 6) and Fe_3_O_4_ was detected as the main corrosion product detected. The worst performance was observed in the copper specimen, as expected from the gravimetric chart (Figure 1). Figure 7 and Figure 8 show a detailed picture of the surface and the cross-section, respectively, of the copper coupon. A corrosion layer 5.14 µm in thickness can be easily identified in Figure 8 and the energy-dispersive X-ray (EDX) analysis confirmed this conjecture. When tested, this corrosion layer possessed a 28.61% oxygen fraction. When comparing points A and B in the figure, a protective corrosion layer (A) that formed on the copper surface may also be observed.

**Figure 9:**
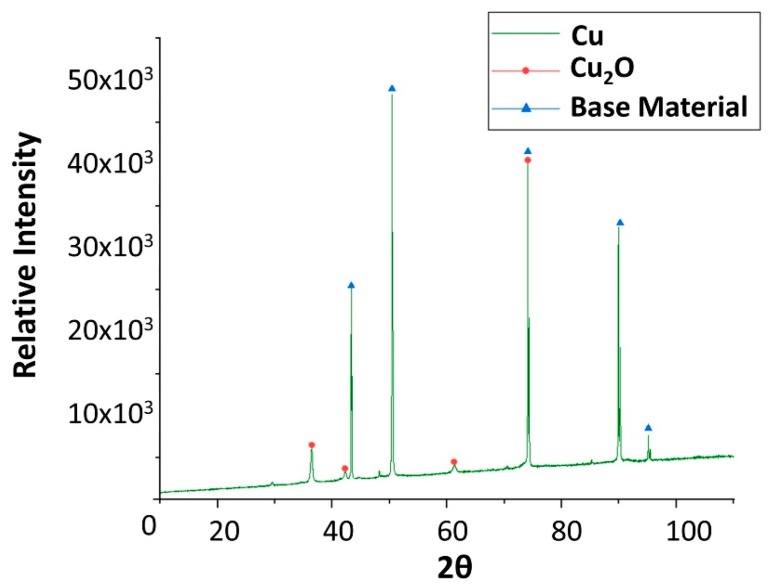
X-ray diffraction analysis of the copper specimen after 4 weeks immersed in solar salt.

Finally, x-ray diffraction (XRD) assays were performed to detect the corrosion that occurred. Only the results obtained from copper and carbon steel specimens were plotted following analysis, as these are the samples attacked by corrosion. XRD carried out on carbon steel (Figure 6) detected magnetite (Fe_3_O_4_) as the main corrosion product. As shown in Figure 9, cuprous oxide (Cu_2_O) was detected as a result of immersion in the solar salt.

All in all, solar salt has proven its compatibility with three out of the four assessed metals. Beginning with a rejection of copper due to its low corrosion resistance, from the other candidates, aluminium and SS304 have shown better corrosion resistance than carbon steel. However, aluminium and SS304 are approximately double and triple the price of carbon steel (AISI 1090), respectively. For that reason, a trade-off between price and the lifespan of the material needs to be considered.

### 2.2. Corrosion Produced by Myo-Inositol (C_6_H_12_O_6_)

**Figure 10:**
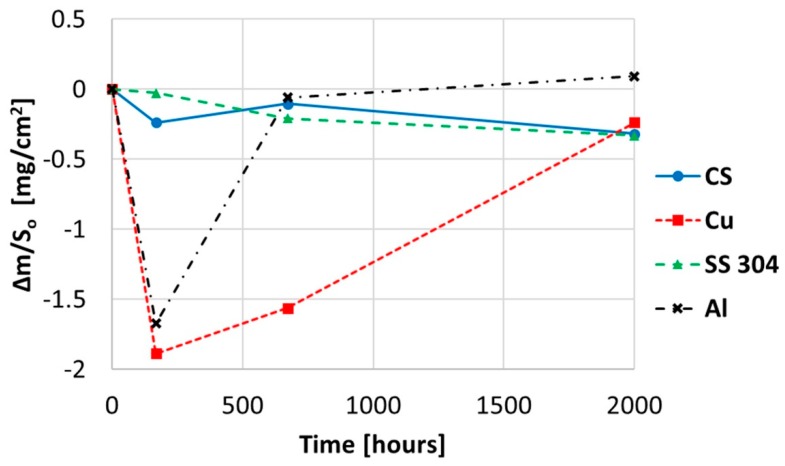
Gravimetric weight loss curve obtained for copper, aluminium, and stainless and carbon steel when immersed in myo-inositol at 250 °C.

The gravimetric weight loss chart when immersing copper, aluminium, and stainless and carbon steel in myo-inositol at 250 °C for 2000 h is plotted in Figure 10. These results show irregular behaviour due to the high viscosity of the molten sugar, which strongly sticks to the coupons. This stacked sugar forms a crystal compound when solid, which can drag off possible corrosion products if it sloughs off itself. 

The gravimetric chart corresponds to the weight lost following the corrosion process. It is important to highlight that the high viscosity found in sugar alcohol represents a significant influence to the cleaning process. 

**Figure 11:**
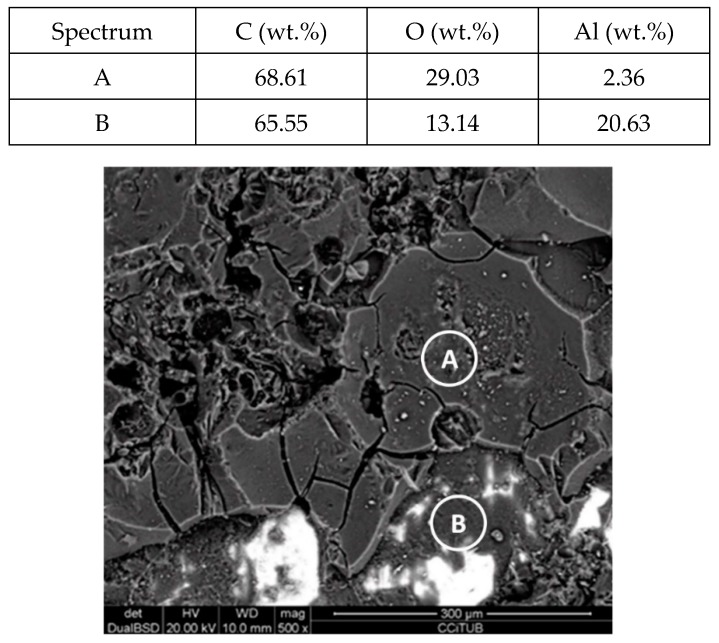
Detail (lower) and EDX analysis (upper) of the aluminium specimen surface after 2000 h immersed in myo-inositol.

**Figure 12:**
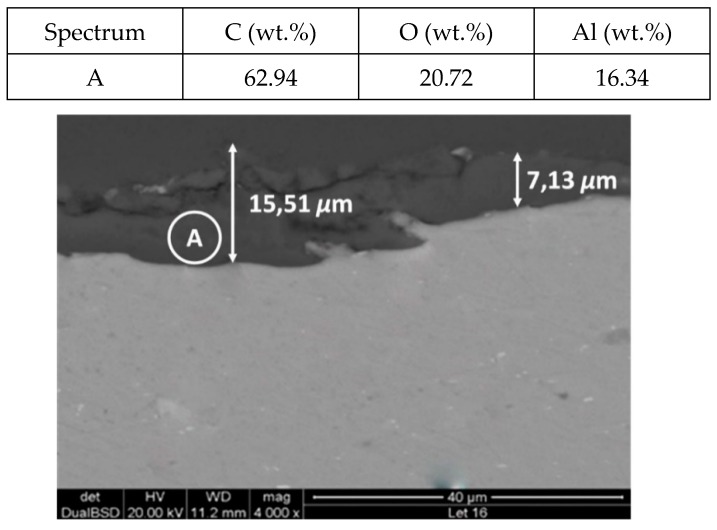
Detail (lower) and EDX analysis (upper) of the aluminium specimen cross-section after 2000 h immersed in myo-inositol.

**Figure 13:**
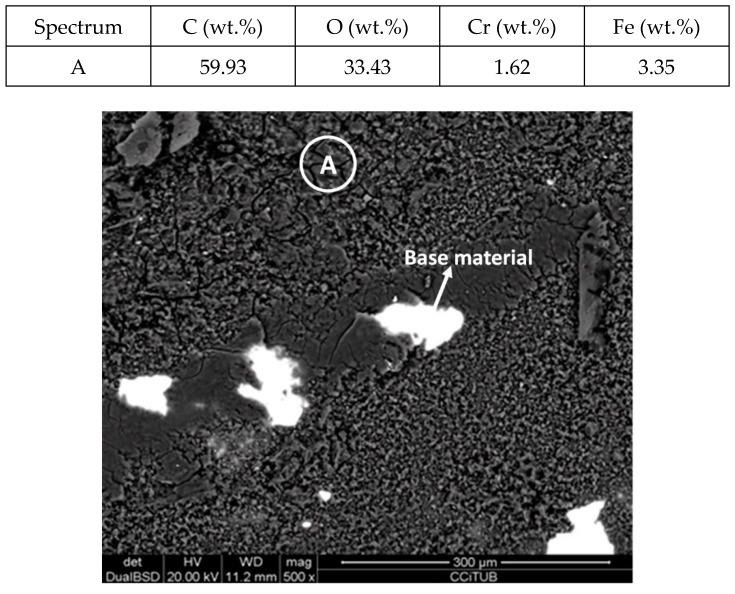
Detail (lower) and EDX analysis (upper) of the stainless steel specimen surface after 2000 h immersed in myo-inositol.

**Figure 14:**
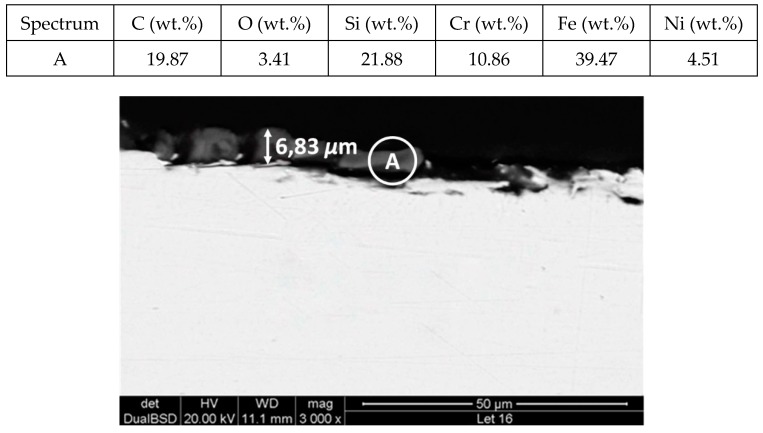
Detail (lower) and EDX analysis (upper) of the stainless steel specimen cross-section after 2000 h immersed in myo-inositol.

**Figure 15:**
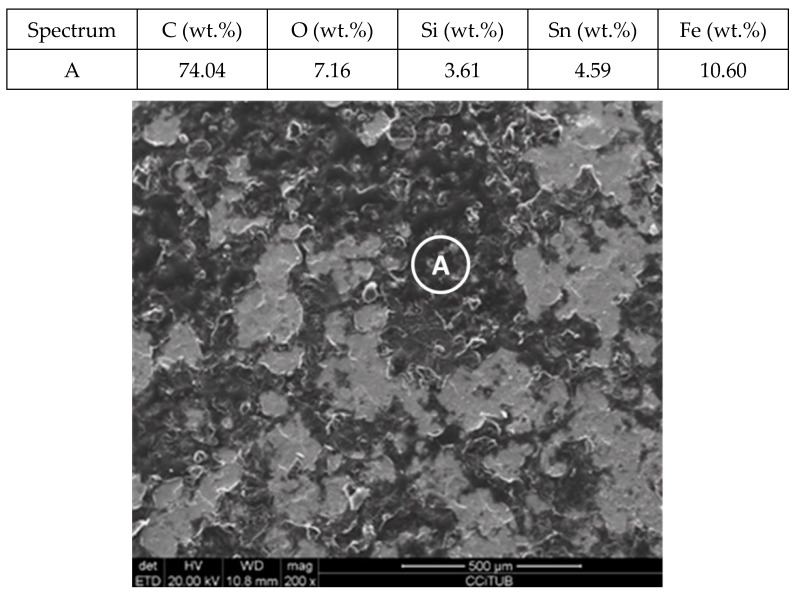
Detail (lower) and EDX analysis (upper) of the carbon steel specimen surface after 2000 h immersed in myo-inositol.

**Figure 16:**
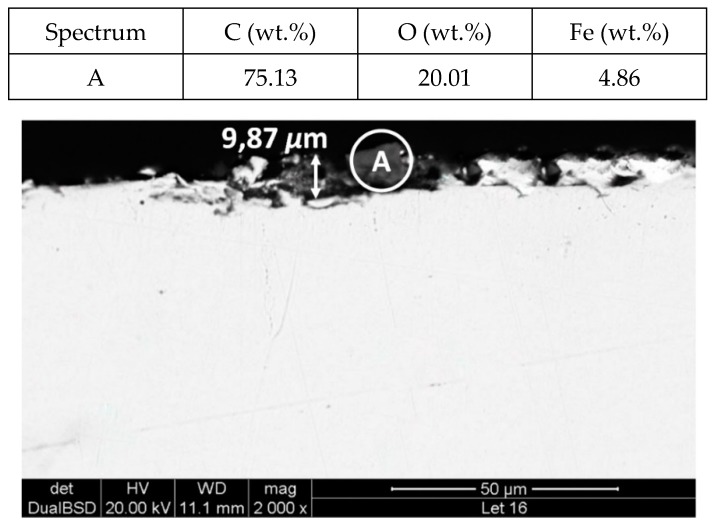
Detail (lower) and EDX analysis (upper) of the carbon steel specimen cross-section after 2000 h immersed in myo-inositol.

**Figure 17:**
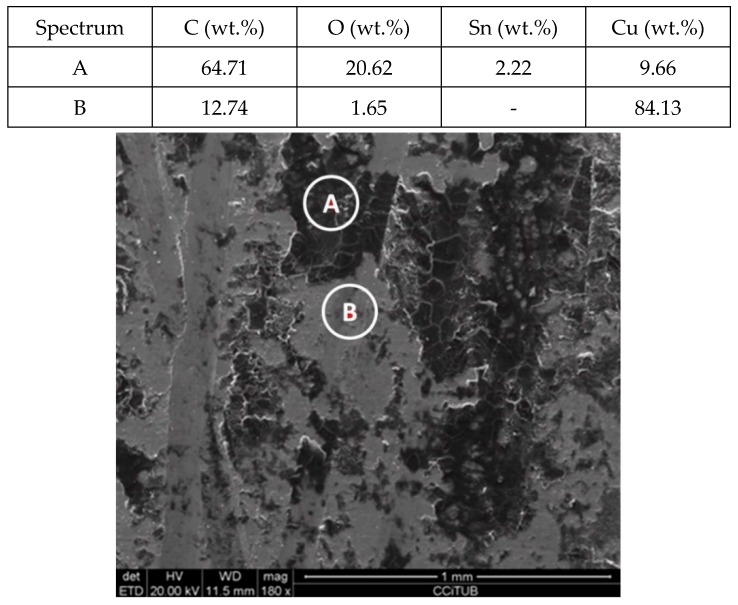
Detail (lower) and EDX analysis (upper) of the copper specimen surface after 2000 h immersed in myo-inositol.

**Figure 18:**
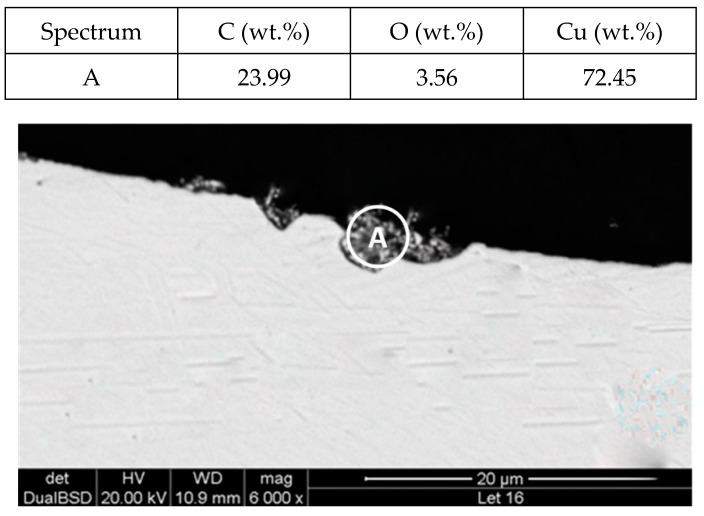
Detail (lower) and EDX analysis (upper) of the copper specimen cross-section after 2000 h immersed in myo-inositol.

Figure 11 and Figure 12 show a thick layer of myo-inositol stacked on the aluminium probe. The spectrums A and B analysed on the aluminium surface probe (Figure 11) indicate a notable presence of the sugar (C wt.%), which can also be visually identified. The sugar layer thickness grows up to 15.51 µm after 2000 h at 250 °C (Figure 12). However, there is no corrosion present on the aluminium specimen. When assessing the stainless steel coupon (Figure 13 and Figure 14), the sugar layer found was thinner compared to the aluminium specimen, probably due to the latter’s ductility, as it makes it easier for the molten myo-inositol to adhere to it. The carbon steel (Figure 15 and Figure 16) and copper (Figure 17 and Figure 18) specimens—unlike the others—show a different morphology, which could suggest some other corrosion products besides the stuck sugar. On both surfaces, a layer of stuck myo-inositol can be appreciated, which seems to be thinner than the one stacked on the aluminium and SS304 probes. 

The cross-section morphology of both coupons, shown in Figure 16 and Figure 18 and corresponding to AISI 1090 and Cu, respectively, confirmed that the layer on the base material is different. Not only is the layer not uniform all over the probe, but the texture is also different, suggesting that on this occasion there is slight corrosion of the specimens. Nevertheless, the corrosion rates shown by each material when immersed in myo-inositol at 250 °C for 2000 h are not very significant (Table 2), with all of them being lower than 0.02 mm per year.

**Figure 19:**
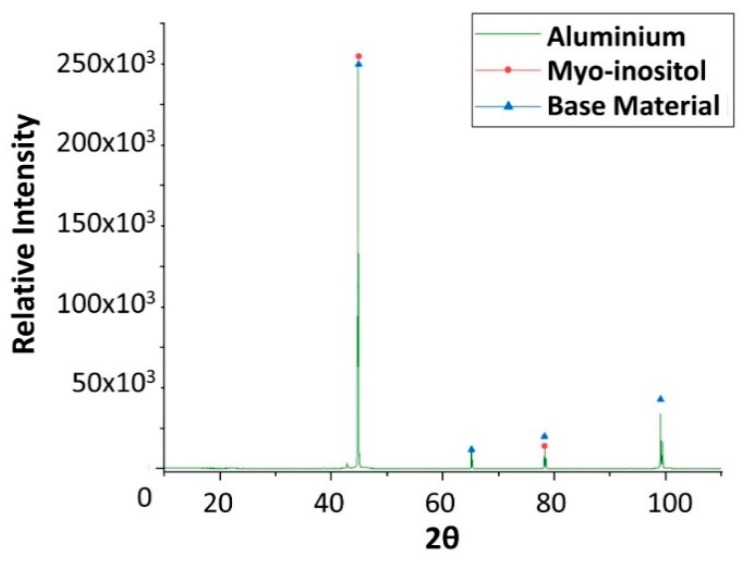
X-ray diffraction analysis of the aluminium specimen after 4 weeks immersed in myo-inositol.

**Figure 20:**
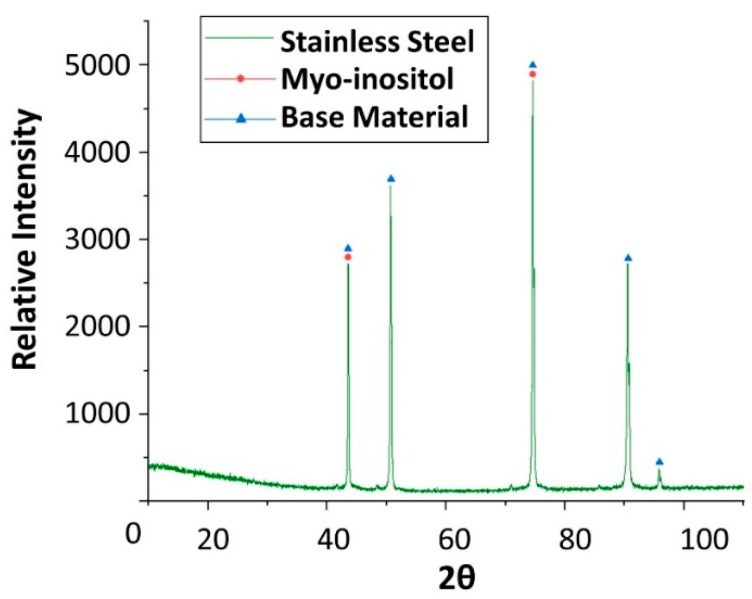
X-ray diffraction analysis of the stainless steel SS304 specimen after 4 weeks immersed in myo-inositol.

**Figure 21:**
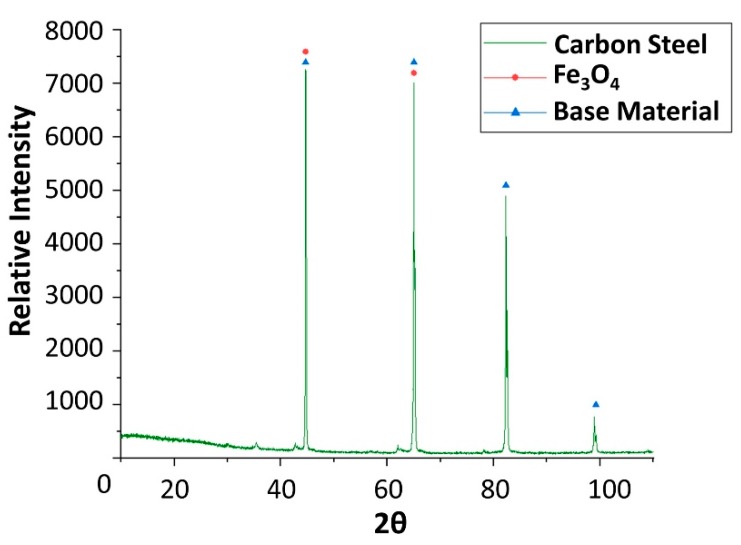
X-ray diffraction analysis of the carbon steel AISI 1090 specimen after 4 weeks immersed in myo-inositol.

**Figure 22:**
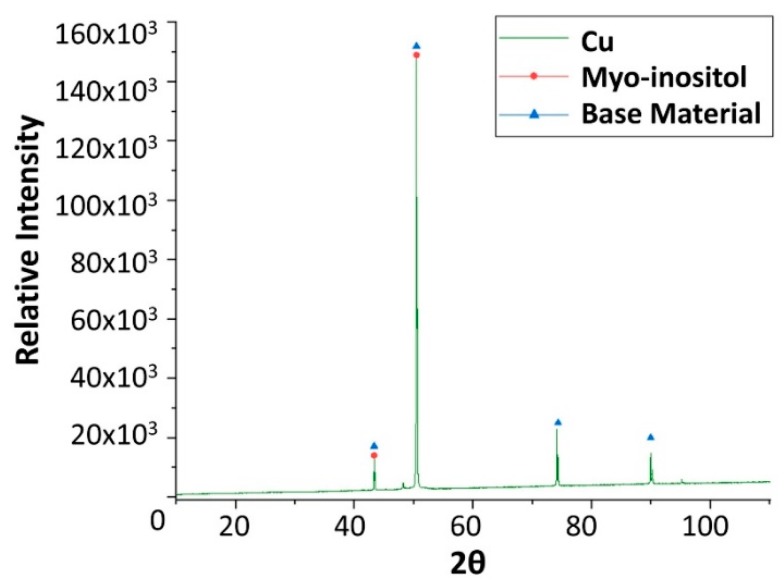
X-ray diffraction analysis of the copper specimen after 4 weeks immersed in myo-inositol.

Finally, x-ray diffraction (XRD) analyses were performed on the specimens after the corrosion test (Figure 19, Figure 20, Figure 21 and Figure 22), confirming the presence of myo-inositol adhered in the materials surface. Figure 19, Figure 20 and Figure 22 show the presence of myo-inositol on the aluminium, SS304 and copper coupons, respectively, asserting that the sugar sticks on the metal probes. In Figure 21, however, a corrosion product of carbon steel (Fe_3_O_4_) may be observed.

All tests performed using myo-inositol appear to discourage the use of this material as a PCM beyond its corrosion rate since the sugar strongly sticks to the metals, making it extremely difficult to manage.

### 2.3. Uncertainties Analysis 

In order to show the impact of the different parameter uncertainties on the results, an uncertainty analysis was performed. This analysis is required to determine and validate the present study. The uncertainties of the measured parameters are shown in Table 3, while the mass loss on every single specimen was calculated using an equation reported in Reference [18] (Equation (1)).

(1) $$\frac{\Delta m}{S_{0}}\;=\;\frac{m_{f}-m_{i}}{S_{0}}\;\;\;\;$$

where *m_i_* is the initial mass of the specimen, *m_f_* is the mass of the same at time t, and *S_0_* is the initial area of the specimen.

Equation (2) allows for an estimation of the uncertainties of the parameters, calculated using the measured ones (Table 4). The uncertainty was estimated for each sample. Table 5 shows the uncertainty in the mass loss of each of the specimens studied, as well as the parameters (area and weight difference) used in the calculation. Equation (2) is given as:

(2) $$W_{R}={\left[{\left(\frac{\partial R}{\partial x_{1}}\cdot w_{x_{1}}\right)}^{2}+{\left(\frac{\partial R}{\partial x_{2}}\cdot w_{x_{2}}\right)}^{2}+\ldots +{\left(\frac{\partial R}{\partial x_{n}}\cdot w_{x_{n}}\right)}^{2}\right]}^{1/2}$$

where *W_R_* is the estimated uncertainty, *R* is the function based on the measured parameters, *x_n_* represents the range of measured parameters, and *w_x_* represents the uncertainties of those measured parameters.

## 3. Materials and Methods 

The corrosive environment used in this research is a eutectic mixture of 40 wt.% KNO_3_ and 60 wt.% NaNO_3_, commonly called solar salt, and myo-inositol (C_6_H_12_O_6_), which is a sugar alcohol regularly used in food industries under the U.S.A NF12/FCCV standard. The first of the selected materials was an inorganic compound: 98% purity KNO_3_ from VWR and Panreac Química provided >99% purity NaNO_3_, while the other studied material was an organic compound, more specifically a sugar alcohol: >98% purity Pentaerythritol from Xia’an lyphar biotech Co. (Xi’an, China). 

**Figure 23:**
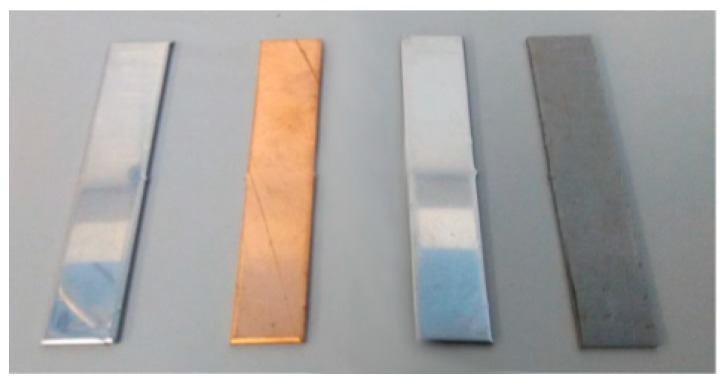
Metal specimens used in the corrosion tests. From left to right: Aluminium, copper, stainless steel (AISI 304), and carbon steel (AISI 1090).

**Figure 24:**
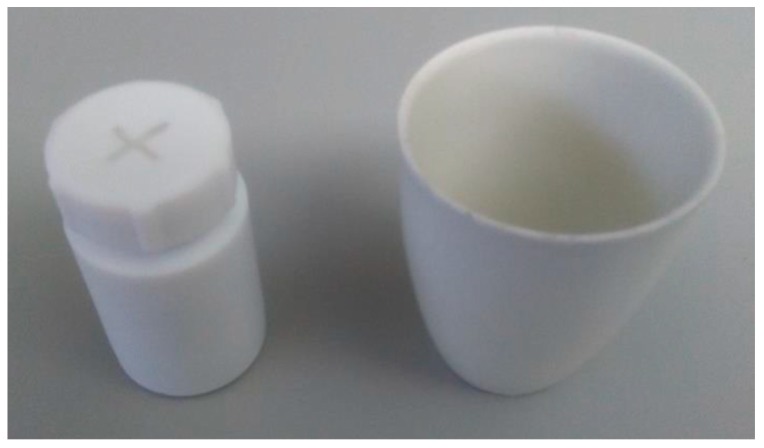
Polytetrafluoroethylene (PTFE) closed crucible and porcelain crucible respectively.

To quantitatively evaluate the corrosion rate, a gravimetric analysis was performed. This assay required an immersion of the different metal samples (Figure 23) into the corrosive environment for 2000 h at 250 °C; the chemical composition of every metal compound can be found in Table 6. The specimens were completely dipped in porcelain or PTFE crucibles (Figure 24), combining every metal specimen with the two different PCMs. The crucibles were kept in a furnace at 250 °C so the PCMs were always in a liquid state. Importantly, the myo-inositol was confined in polytetrafluoroethylene (PTFE) closed crucibles following the advice from previous works with this compound [9]. 

The specimens used in the corrosion test were measured in order to determine the area in contact with the molten salt. The dimensions of the analysed samples in the gravimetric corrosion tests were 27 mm × 10 mm × 2 mm. The dimensions of each sample were measured using an electronic calibre, as well as weighed using an analytical balance with a 0.00001 g responsiveness (Mettler Toledo AG135). Once the samples were removed, coupons were cooled slowly and then dried and weighed. The mass gained over time was calculated using Equation (1). The gravimetric curve is completed using this value (Y axis) and time of exposure (X axis). In this study, the total time exposed was 2000 h and the samples were taken and measured at 100, 1000 and 2000 h.

During the drying (samples were cleaned after immersion using hot water in order to remove the salt or sugar remaining) and handling processes, specimens can undergo spallation and therefore loss of corrosion layers due to variation in the thermal expansion coefficients of different oxides formed on the surface of the steel. This phenomenon could produce non-accurate values during gravimetric testing. In order to avoid this uncertainty, the other way to evaluate the gravimetric corrosion involves the opposite evaluation. 

In this alternative method, the weight lost in the steel sample after removing the corrosion layer produced during the corrosion process is measured. The methodology proposed by the ASTM standard (G1-03) [19] to evaluate corrosion is widely used in many fields. 

The procedure involves corroded metal immersed in a cleaning solution that reacts with the oxide layer and depends on the nature of the base material. The ASTM standard proposes different solutions and thermal treatments, thereby ensuring the removal of the corrosion products only. In this study, three different dissolutions were needed for this test: A 10 vol.% sulphuric acid (H_2_SO_4_) dissolution for cleaning the copper specimens, a dissolution of 50 ml hydrochloric acid (HCl), 1 g antimony trioxide (Sb_2_O_3_), and 2.5 g stannous chloride (SnCl_2_) to clean both the steel samples, and nitric acid (HNO_3_ 96%) to clean the aluminium probe.

Finally, when removed from the corrosive medium, the metal specimens were analysed by scanning electron microscopy (SEM) and x-ray diffraction (XRD) to detect the corrosion produced, and therefore extra specimens were required. All in all, 40 metal samples—10 per each metal—were required. The XRD device used was the PANalytical X´Pert PRO model, and measures were taken from 5 to 120° with a step size of 0.017°, while the SEM model used was the Quanta 250, Thermofisher.

## 4. Conclusions

The presented work performed a corrosion study of four different metals in solar salt (40 wt.% KNO_3_/60 wt.% NaNO_3_) and myo-inositol (C_6_H_12_O_6_), with the aim of using those as PCMs to be applied as thermal energy storage materials in linear Fresnel CSP plants. The study comprised of assays with aluminium, stainless steel (AISI 304), carbon steel (AISI 1090), and copper.

The results obtained when performing the corrosion assays showed that solar salt was compatible with aluminium, stainless steel and carbon steel. However, the corrosion rate of the copper sample was high enough (0.04437 mm/year) to justify discarding it. With regard to the three plausible options, the corrosion rate of carbon steel (AISI 1090) was higher than the other candidates, but still was acceptable depending on the application lifespan.

The results shown by the corrosion tests performed with myo-inositol did not allow for an accurate conclusion concerning the corrosion resistance of the four metals, since the sugar strongly stuck to the metal coupons. It has to be taken into account that despite the closed atmosphere for the experiments with myo-inositol, the oxygen was still able to oxidize the myo-inositol, combusting the sugar and leaving nothing but ashes and the myo-inositol stuck on the probe. All in all, the use of myo-inositol as a PCM has to be discouraged.

## Figures and Tables

**Table 1:** Phase change material (PCM) candidates with melting points between 210 °C and 270 °C which successfully completed thermal characterization.

Material	Price [€/kg]	Melting Temperature [°C]	Melting Enthalpy [J/g]	Ref.
**Myo-inositol (C_6_H_12_O_6_)**	8–10	220	190–223	[9,17]
**Solar salt (40 wt.% KNO_3_/ 60 wt.% NaNO_3_)**	33	222	94–100	[9,11]

**Table 2:** Corrosion rate of the different materials in solar salt and myo-inositol.

	Solar Salt	Myo-inositol
	Corrosion Rate [mm/year]	
**Aluminium**	0.0015	0.0150
**Stainless Steel AISI 304**	0.0012	0.0182
**Carbon Steel (AISI 1090)**	0.0111	0.0178
**Copper**	0.0444	0.0116

**Table 3:** Uncertainties of the different parameters involved in the analyses of the present study.

Parameter	Units	Sensor	Accuracy
**Weight**	g	Mettler Toledo AG135	±0.00001 g
**Length**	mm	Digital caliper	±0.01 mm
**Width**	mm	Digital caliper	±0.01 mm
**Thickness**	mm	Digital caliper	±0.01 mm

**Table 4:** Parameters calculated from the measurements.

	Weight Difference	Area	Mass Loss
	[g]	[mm^2^]	[g/mm^2^]
**Carbon Steel (AISI 1090)**	1.33 × 10^−3^	569.125	2.337 × 10^−6^
	1.05 × 10^−3^	658.436	1.595 × 10^−6^
	1.33 × 10^−3^	667.290	1.993 × 10^−6^
**Copper**	1.55 × 10^−3^	595.823	2.601 × 10^−6^
	3.18 × 10^−3^	640.614	4.964 × 10^−6^
	5.62 × 10^−3^	627.983	8.949 × 10^−6^
**Stainless steel (AISI 304)**	0.18 × 10^−3^	552.278	3.259 × 10^−6^
	0.18 × 10^−3^	656.986	2.740 × 10^−6^
	0.14 × 10^−3^	638.872	2.191 × 10^−6^
**Aluminium**	0.3 × 10^−3^	569.180	5.271 × 10^−6^
	0.21 × 10^−3^	643.462	3.264 × 10^−6^
	0.06 × 10^−3^	647.667	9.264 × 10^−6^

**Table 5:** Estimated uncertainties of the calculated parameters.

Uncertainty	Weight Difference		Area		Mass Loss	
Steels	[± g]	[± %]	[± mm^2^]	[± %]	[± g/mm^2^]	[± %]
**Carbon Steel (AISI 1090)**	0.00001	0.752	0.069	0.012	1.757 × 10^−8^	0.752
	0.00001	0.952	0.069	0.011	1.519 × 10^−8^	0.953
	0.00001	0.752	0.069	0.010	1.499 × 10^−8^	0.752
**Copper**	0.00001	0.645	0.069	0.012	1.679 × 10^−8^	0.645
	0.00001	0.314	0.069	0.011	1.562 × 10^−8^	0.315
	0.00001	0.178	0.069	0.011	1.595 × 10^−8^	0.178
**Stainless steel (AISI 304)**	0.00001	5.556	0.069	0.013	1.81 × 10^−8^	5.556
	0.00001	5.556	0.069	0.011	1.522 × 10^−8^	5.556
	0.00001	7.143	0.069	0.011	1.565 × 10^−8^	7.143
**Aluminum**	0.00001	3.333	0.069	0.012	1.757 × 10^−8^	3.333
	0.00001	4.762	0.069	0.011	1.554 × 10^−8^	4.762
	0.00001	16.667	0.069	0.011	1.544 × 10^−8^	16.667

**Table 6:** Chemical composition of the materials tested in molten salts.

Steels	Weight (%)									
	Al	Mn	Ni	Cr	P	C	S	Fe	Cu	Mo
**Stainless steel (AISI 304)**	-	1.7	8.04	18.28	-	-	-	Balance	-	0.27
**Carbon steel (AISI 1090)**	-	0.6–0.9	-	-	0.04	0.85–0.98	0.05	Balance	-	-
**Aluminium**	100	-	-	-	-	-	-	-	-	-
**Copper**	-	-	-	-	-	-	-	-	100	-
